# A Scalable Synthesis of Ag Nanoporous Film As an Efficient SERS-Substrates for Sensitive Detection of Nanoplastics

**DOI:** 10.1021/acs.langmuir.4c01671

**Published:** 2024-08-05

**Authors:** Rafael Villamil Carreón, Ana G. Rodríguez-Hernández, Laura E. Serrano de la Rosa, Ma. Estela Calixto, J.J. Gervacio-Arciniega, Siva Kumar Krishnan

**Affiliations:** † Facultad de Ciencias Físico Matemáticas, Benemérita Universidad Autónoma de Puebla, Av. San Claudio y Av. 18 sur., Puebla, Puebla 72570, México; ‡ CONAHCyT-Centro de Nanociencias and Nanotecnología, Universidad Nacional Autónoma de México, Km 107 Carretera Tijuana-Ensenada Apdo Postal 14, Ensenada, Baja California 22800, México; Δ Instituto de Física, Benemérita Universidad Autónoma de Puebla, Av. San Claudio y Blvd. 18 Sur, Col. San Manuel, Ciudad Universitaria, Puebla, Puebla 72570, México; # CONAHCyT—Facultad de Ciencias Físico Matemáticas, Benemérita Universidad Autónoma de Puebla, Apdo. Postal J-48, Puebla, Puebla 72570, México; § CONAHCyT—Instituto de Física, Benemérita Universidad Autónoma de Puebla, Av. San Claudio y Blvd. 18 Sur, Col. San Manuel, Ciudad Universitaria, Puebla, Puebla 72570, México

## Abstract

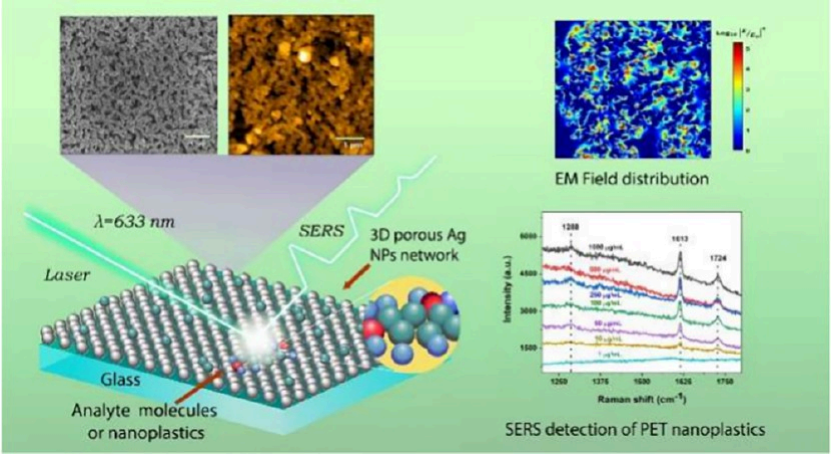

Nanoplastics pollution has led to a severe environmental crisis because of a large accumulation of these smaller nanoplastic particles in the aquatic environment and atmospheric conditions. Detection of these nanoplastics is crucial for food safety monitoring and human health. In this work, we report a simple and eco-friendly method to prepare a SERS-substrate-based nanoporous Ag nanoparticle (NP) film through vacuum thermal evaporation onto a vacuum-compatible deep eutectic solvent (DES) coated growth substrate for quantitative detection of nanoplastics in environmental samples. The nanoporous Ag NP films with controlled pores were achieved by the soft-templating role of DESs over the growth substrate, which enabled the self-assembly of deposited Ag NPs over the surface of DES. The optimized nanoporous Ag substrate provides high sensitivity in the detection of analyte molecules, crystal violet (CV), and rhodamine 6G (R6G) with a limit of detection (LOD) up to 1.5 × 10^–13^ M, excellent signal reproducibility, and storage stability. Moreover, we analyzed quantitative SERS detection of polyethene terephthalate (PET, size of 200 nm) and polystyrene (PS, size of 100 nm) nanoplastics with an LOD of 0.38 and 0.98 μg/mL, respectively. In addition, the SERS substrate efficiently detects PET and PS nanoplastics in real environmental samples, such as tap water, lake water, and diluted milk. The enhanced SERS sensing ability of the proposed nanoporous Ag NP film substrate holds immense potential for the sensitive detection of various nanoplastic contaminants present in environmental water.

## Introduction

The pollution caused by the nanoplastics particle (size less than ∼1000 nm) is one of the most concerning environmental problems, which poses a high risk to the atmosphere and human health.^1^ Thus, the accurate detection of nanoplastics is essential for various fields such as food safety and environmental monitoring.^2^ Recently, research efforts have been directed toward developing analytical methods to detect smaller nanoplastic contamination in the environment.^2−4^ However, sensitive detection of nanoplastics with low concentration levels in complex environmental samples remains elusive due to the smaller size fraction of particulate nanoplastics.^5^ Surface-enhanced Raman scattering (SERS) spectroscopy has emerged as a powerful, nondestructive spectroscopy technique that offers sensitive identification of analytes with high sensitivity and the ability to identify molecular fingerprints.^6−8^ To achieve high sensitivity and good signal reproducibility as well as stability of the SERS substrates, various strategies have been developed.^8−10^ In particular, “hotspot” engineering in the gaps of plasmonic NPs such as Ag, Au, and Cu,^11−13^ designing three-dimensional (3D) plasmonic nanostructures,^14,15^ plasmonic NPs on 2D materials such as graphene, MoS_2_, Mxane, and their heterostructures have been widely studied.^16−18^ Nevertheless, the fabrication of a SERS substrate with maximum “hotspots” density for a highly reproducible signal and implementation of the plasmonic NP substrates in direct detection of target analytes in complex environments is still elusive.^10^

Recently, the development of porous three-dimensional (3D) substrates has received special attention for efficient SERS detection because of their tunable LSPR properties ranging from ultraviolet (UV) to near-infrared (NIR), high surface area, and strong confinement of electromagnetic (EM) field strength within the pores, which provide optimum sites to interact with the analyte molecules.^19,20^ Specifically, porous Ag- or Au-containing nanometric pores have been demonstrated to exhibit remarkably enhanced SERS activity for a variety of analyte molecules.^21−23^ For instance, Lim et al.^21^ showed that mesoporous Ag films exhibited greatly enhanced molecular interaction, and a SERS enhancement factor (EF) on the order of 10^7^ to 10^8^ was achieved. Also, La and co-workers,^22^ have recently demonstrated that the SERS substrate based on nanoporous Au alloys showed a SERS EF up to 10-times greater than that of the monolayer Au NPs and exhibited superior sensitivity down to 1 fM concentrations of analyte molecules. Such nanoporous structures have been prepared through different physical and chemical routes such as chemical synthesis and self-assembly,^24^ Langmuir–Blodgett,^25^ electron beam lithography,^11^ chemical dealloying,^26^ electrochemistry,^27,28^ treatment of oxygen plasma of thermally deposited films,^29^ and dewetting of metallic films^30^ to create a porous 3D framework. All of these methods are time-consuming and costly and have difficulty in generating 3D plasmonic nanostructures. Alternatively, the vacuum thermal evaporation process is emerging as a low-cost and commercialized deposition technique for creating high-quality thin films on a solid substrate.^31^ However, to achieve films consisting of nanoparticles with varied size, shape, and distribution, self-assembly is highly difficult. Therefore, research efforts have been directed toward the integration of different vacuum pressure compatible liquid/solvent-coated substrates in the thermal evaporation process.^32,33^ In general, metal vapors by resistive heating will travel a long mean free path to condense onto the liquid surfaces to generate discontinuous porous networks, which is completely distinct from the films obtained by a conventional thermal evaporated process.^33^

Deep eutectic solvents (DESs) are a new class of ionic liquids (ILs), which have gained considerable attention as green solvents with preorganized solvent structures for many applications.^34,35^ DESs are eutectic mixtures, which are generally formed by the mixing of pairs of hydrogen bond donors and acceptors with certain molar fractions, resulting in significant depression of melting points.^35^ These solvents are a class of versatile green medium, which can be prepared easily at a relatively low cost and exhibit unique properties such as ionic character, biodegradability, nontoxicity, high ionic conductivity, tunable viscosity, and polarity. Owing to their unique properties, DES has been widely used as a potential green solvent for many promising applications such as an electrolyte for metal ion batteries, in CO_2_ capture, in biocatalysis, and in the synthesis of functional materials.^36,37^ Moreover, DES exhibits extremely low vapor pressure in the range of 35.32 to 60.77 Pa below 343.15 K,^38^ and it withstands high vacuum pressure, which makes it ideal for vacuum deposition conditions. In addition, owing to their extended hydrogen bonding structure, DES has been used as a green medium for metallic nanoparticle synthesis^39^ and self-assembly of metal NPs over the DES surface.^40,41^ For instance, DESs have been integrated with vapor phase deposition processes such as atomic layer deposition (ALD) to perform catalytic surface reactions.^42,43^ We have also recently demonstrated the successful integration of DESs with the thermal evaporation process to prepare Ag–Au bimetallic nanoparticle films.^44^ However, preparing nanoporous Ag over the DES surface using thermal evaporation is a challenging task.

In this present study, we demonstrate a simple and low-cost synthetic strategy by successful integration of DES solvent with a vacuum thermal evaporation process to prepare nanoporous Ag NP films as a reproducible SERS substrate for nanoplastics detection. These nanoporous Ag NP films have a robust nanoporous network structure with high surface area, which effectively generates a high density of “hotspots”. Benefiting from the maximum density of “hotspots”, the fabricated nanoporous SERS substrate exhibits high sensitivity in the detection of analyte probe molecules such as CV and R6G molecules with an LOD as low as 1.5 × 10^–13^ M. In addition, the SERS substrate demonstrates the capability of the detection of nanoplastic particles such as polyethene terephthalate (PET, size of ∼200 nm) and commercial polystyrene nanospheres (PS, size of ∼100 nm) in complex environmental media such as tap water, lake water, and diluted milk samples with excellent selectivity. The proposed strategy provides a new platform for the development of low-cost and highly reproducible nanoporous substrates for SERS analysis of nanoplastic contamination in water samples.

## Experimental Section

### Preparation of Deep Eutectic Solvent (DES)

To prepare DES, choline chloride (ChCl) was first dried at 90 °C in an oven to ensure that ChCl is completely dry. Urea and ChCl were mixed in a molar ratio of 2:1 and hermetically sealed, and the mixture was heated in an oven at 90 °C until a clear, homogeneous liquid was formed. The viscous liquid was then cooled to room temperature and stored for later use.

### Preparation of Nanoporous Silver NPs Films

**Scheme 1:**
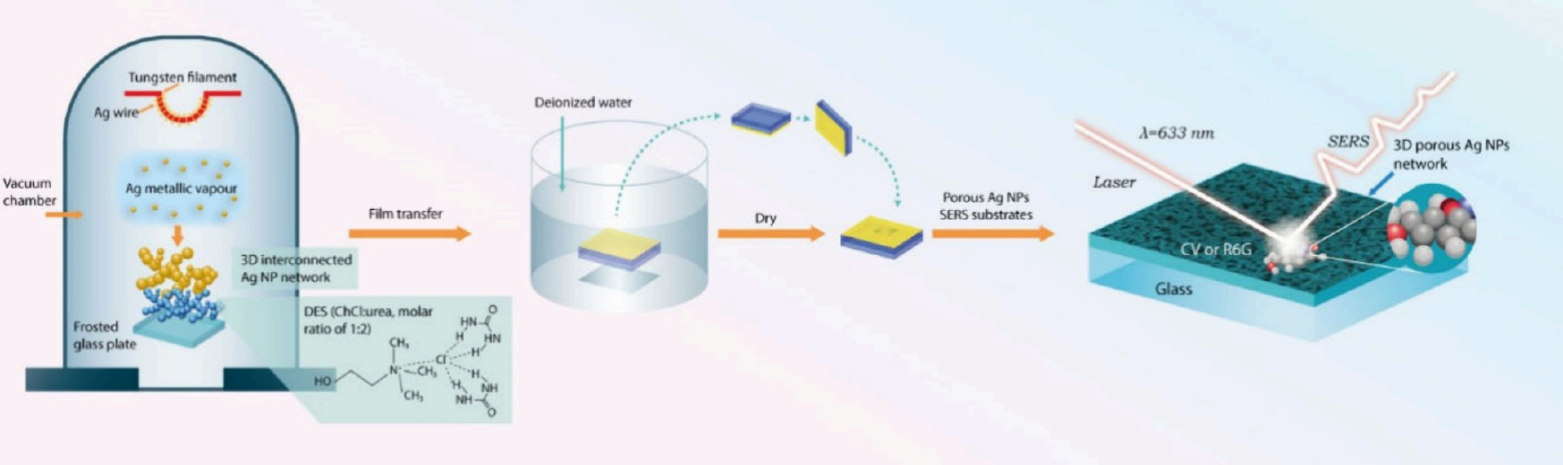
Schematic Illustration of Steps Involved in the Preparation of Nanoporous Ag NP Film Substrates Using Vacuum Thermal Evaporation onto the Surface of DESs

First, glass substrates (size of 2.5 × 2.5 cm^2^) were cleaned with trichloroethylene, acetone, and ethyl alcohol for 15 min in a sonic bath and dried well. Then, the DES solvent was uniformly coated onto the glass substrate with ∼1 mm thickness and was mounted inside the vacuum chamber for the thermal evaporation process. The silver wire (purity of 99.99%) was thermally evaporated by tungsten filament onto the DES-coated substrates with varied pressures of P1 = 10^–2^ mbar, P2 = 10^–3^ mbar, P3 = 10^–4^ mbar, and P4 = 2 × 10^–4^ mbar and an applied current of 2, 4, 8, and 12 A, respectively. Subsequently, each sample was gently covered with another clean glass slide on the surface of the Ag film, and then the sandwich formed with the glass slides was kept for a few minutes so that the film could better adhere to the clean glass slide, as shown in Scheme 1. Finally, the sandwich structure was immersed in DI water for 1 min and gently sprayed with the help of a micropipette to remove DES, and the substrates were allowed to dry at room temperature.

### SERS Substrate Preparation

SERS substrates were prepared by depositing 25 μL of an aqueous crystal violet solution (CV, 10^–6^ M) onto the different Ag NP film substrates and allowed to dry under ambient conditions. Raman spectra were obtained by using a He–Ne laser with an excitation wavelength of 633 nm. For all samples, the laser spot was set to 4 μm under a 10× objective lens, the power density was 2.7 mW, and the time of signal acquisition was set to 2 s and was used to record SERS spectra.

### SERS Detection of PET Nanoplastics

The PET nanoplastic particles with an average size of about 200 nm were synthesized by following the protocol of our previous work.^45^ Specifically, 1 g of PET particles was dissolved in 10 mL of concentrated trifluoroacetic acid solution (TFA, 90% v/v) at 50 °C and stirred for 2 h until complete dissolution overnight. Then, the above solution was precipitated by adding 10 mL of diluted TFA (20% v/v) under vigorous stirring and kept for 2 h. The suspension was centrifuged at 2500 rpm for 1 h to separate the nanoplastic particles. The commercial polystyrene (PS) nanospheres with an average size of 100 nm were purchased from Sigma-Aldrich, Mexico. For SERS detection of nanoplastics, first, the dispersion of PET and PS with varied concentrations was prepared in DI water. Then, 25 μL of nanoplastic dispersion was drop-casted onto the obtained nanoporous Ag NP substrates and allowed to dry naturally before the SERS measurements.

### SERS Detection in Real Samples

For real-sample analysis, tap water and river water were collected from the lab and nearby lake. The milk samples were prepared by dilution of purchased milk from a local store (1 mL of samples in 10 mL of DI water). Then, 1 mL (100 μg/mL) of PET and PS nanosphere dispersions with different concentrations were spiked into 10 mL of DI water, tap water, river water, and diluted milk. After that, 25 μL of PET and PS dispersion was deposited onto the SERS substrates and then dried overnight to perform the SERS analysis.

## Results and Discussion

### Synthesis and Characterization of Nanoporous Ag NP Films

Nanoporous Ag NP films were prepared by vacuum thermal evaporation of Ag wire onto a glass substrate coated with a ChCl/urea (molar ratio 1:2) derived deep eutectic solvent (DES), as illustrated in Scheme 1. First, DES was uniformly coated onto the glass substrate and placed inside the thermal evaporation chamber. Subsequently, nanoporous Ag NP films were formed by evaporating a Ag wire over the DES-coated substrate using a thermal evaporation process. The DES surface functioned as a stabilizing soft-templating solvent, facilitating the formation of self-assembled Ag NPs and their nanoporous structure.^40,41^ The nanoporous Ag NP films deposited over DES were subsequently transferred onto another glass substrate to form a sandwich structure, followed by washing to remove the DES. To optimize the morphology and porosity of the Ag NP films, varied deposition pressures of 1 × 10^–2^, 1 × 10^–3^, 1 × 10^–4^, and 2 × 10^–4^ mbar were employed. The samples obtained from these pressures are designated as Ag–P1, Ag–P2, Ag–P3, and Ag–P4, respectively. Additionally, the current applied for Ag wire evaporation varied between 2 and 12 A (Figure S1, Supporting Information). The results demonstrate that using a current of 4 A and a low pressure of 2 × 10^–4^ mbar resulted in uniform deposition of nanoporous Ag NP films over the DES surface.

**Figure 1:**
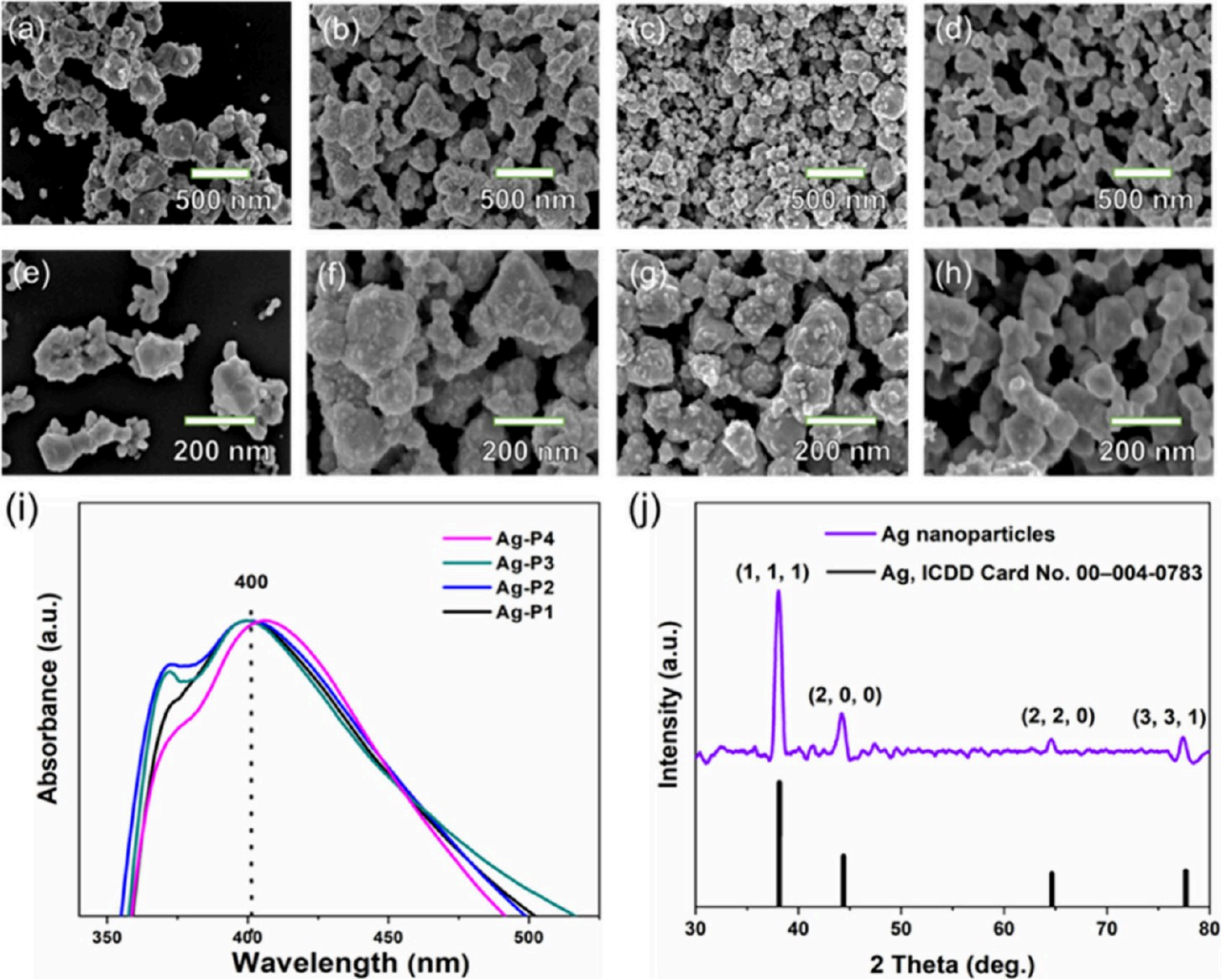
SEM images of the Ag NPs films obtained by thermal evaporation onto DES (ChCl/urea, ratio molar 1:2) with different deposition pressures with constant current (4 A). (a, e) 1 × 10^–2^ mbar, (b, f) 1 × 10^–3^ mbar (c, g) 1 × 10^–4^ mbar, and (d, h) 2 × 10^–4^ mbar, respectively. (i) UV–vis absorption spectra of Ag NPs films obtained at different pressures. (j) XRD pattern of Ag nanoparticles film obtained at 2 × 10^–4^ mbar.

Figure 1 depicts the field emission-scanning electron microscope (FE-SEM) images of Ag NP films deposited under various deposition pressures with a constant applied current of 4 A. At higher pressures (1 × 10^–2^ mbar), the Ag NP films exhibit aggregated particles forming large, crystal-like structures with average particle size of 186 ± 11 nm (Figure 1a,e and Figure S2). Decreasing the pressure to 1 × 10^–3^ and 1 × 10^–4^ mbar, the deposited Ag NP films are mainly composed of self-assembled larger agglomerated Ag particles, with nonuniform pores between them (Figure 1b,c,f,g). Interestingly, deposition at a lower pressure (2 × 10^–4^ mbar) yields self-assembled Ag NPs with relatively smaller particle sizes (107 ± 2.9 nm), eventually forming nanoporous three-dimensional (3D) network-like structures. Upon close observation, it was observed that Ag NPs undergo collapse and transform into irregular spherical particles, which then self-assemble to form uniform nanoporous Ag NP structures composed of an interconnected ligament network (Figure 1d,h). Notably, the particle size decreased from 186 ± 11 nm to 107 ± 2.9 nm upon lowering the deposition pressure (Figure S2). These results indicate that a lower pressure is necessary to produce smaller Ag NPs and subsequently form well-connected nanoporous network structures with uniform pores.

The aforementioned results indicate that achieving nanoporous Ag NP films on the DES surface requires low deposition pressures. Previous studies have shown that increasing deposition pressure during vacuum thermal evaporation can cause metal ion vapors to coalesce, leading to the formation of larger nanoparticles with varied shapes at the liquid interface.^31^ Additionally, it was shown that lowering pressure can reduce the kinetic energy of sputtered metal atoms, restricting their lateral movement at the liquid surface.^46^ On the other hand, lowering pressure can decrease the viscosity of the DES due to enhanced viscous dissipation and expansion of the hydrogen bonding network within the ChCl–urea components of DES.^47^ Such viscosity changes at the DES surface can influence long-range van der Waals interactions between DES and metallic NPs, impacting subsequent self-assembly of Ag NPs.^40,48^ Consequently, deposition at higher pressures (Ag–P1, Ag–P2) may cause Ag atoms to diffuse partially into the bulk DES, promoting self-assembly into agglomerated particles. In contrast, lower pressure (2 × 10^–4^ mbar) reduces the kinetic energy loss of Ag metal vapors, allowing most to settle on the DES surface. This process favors growth and self-assembly of deposited Ag atoms, resulting in the formation of nanoporous Ag NP films. To ascertain the role of DES in forming nanoporous NP films, deposition experiments were conducted on bare glass slides without a DES coating. The results demonstrated the formation of smooth films instead of nanoporous structures (Figure S3), underscoring the critical role of DES in the formation of nanoporous structure. Moreover, supporting experiments were conducted by replacing ChCl/urea based DES with other combinations such as ChCl/malonic acid (molar ratio 1:1) and ChCl/ethelene glycol (molar ratio of 1:4) is displayed in Figure S4. The result showed the formation of porous Ag nanofoam-like films and Ag NP films with nonuniform distribution (Figure S4a,b). These results unambiguously confirm the key role of DES for the formation of nanoporous Ag NP films.

The optical absorption of Ag NP films deposited under different pressures was recorded by using UV–vis spectroscopy, as shown in Figure 1i. As depicted in Figure 1i, the Ag–P1 sample exhibited a broad surface plasmon resonance (SPR) peak centered around 400 nm, with a lower intensity peak in the vicinity of 360 nm. These observed broad and asymmetric peaks suggest the presence of Ag NPs with varying sizes and shapes, possibly indicating the aggregation of Ag NPs into larger anisotropic structures.^49,50^ However, the Ag–P2, Ag–P3, and Ag–P4 samples showed a further broadening of the SPR peak and a red shift toward longer wavelengths. These changes are likely because of the formation of a network of smaller Ag nanoparticles within a nanoporous network, as reported previously.^49^ The crystalline structure of the Ag NP films was confirmed through X-ray diffraction (XRD) analysis, as shown in Figure 1j. The XRD pattern of the Ag–P4 film revealed characteristic peaks at 38.12°, 44.3°, 64.42°, and 77.45°, which are indexed to the (111), (200), (220), and (311) planes of the face-centered cubic (fcc) structure of silver NPs (ICDD card no. 00-004-0783).^49^ The presence of these well-defined peaks in the XRD patterns indicates that the nanoparticles synthesized by our method have an excellent crystalline nature and purity.

**Figure 2:**
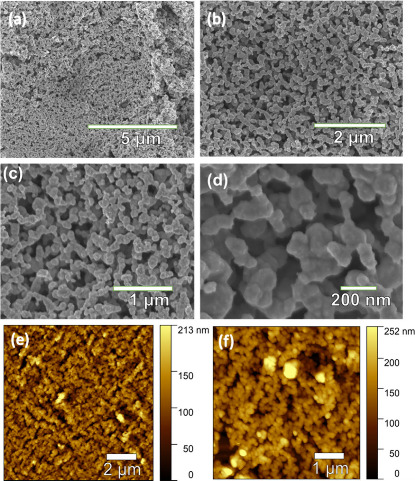
(a, b) Low and (c, d) high magnification SEM images of the as-obtained nanoporous Ag NP film. (e, f) AFM topographic images of nanoporous Ag NP films.

Figure 2a–d displays FE-SEM images of Ag NP films obtained at lower deposition pressures (Ag–P4). As shown in Figure 2a and b, the Ag NPs appear nearly spherical and agglomerate into a 3D nanoporous network structure with uniform pore sizes. Detailed examination at high magnification in SEM images (Figure 2c,d) reveals the formation of interconnected 3D networks comprising Ag NPs interconnected with each other to form nanoporous structures. Furthermore, atomic force microscopy (AFM) images corroborate the formation of uniform nanoporous Ag films with uniform thickness and pore sizes (Figure 2e,f), aligning well with the SEM observations. These findings strongly indicate that the thermal deposition of Ag at lower pressures facilitates the formation of a 3D nanoporous network structure of Ag NPs. Analysis of pore sizes through line-profile measurements in AFM topographic images shows that the average pore size of Ag–P4 is about 300.2 ± 8.8 nm (Figure S5). Notably, the Ag NP films obtained at higher pressures (Ag–P1, Ag–P2, and Ag–P3) showed relatively lower average pore sizes of about 153 ± 6.3, 241.3 ± 8.1, and 279.1 ± 8.1 nm, respectively (Figure S6).

### Optimization of SERS Performance of Ag NP Film Substrates

**Figure 3:**
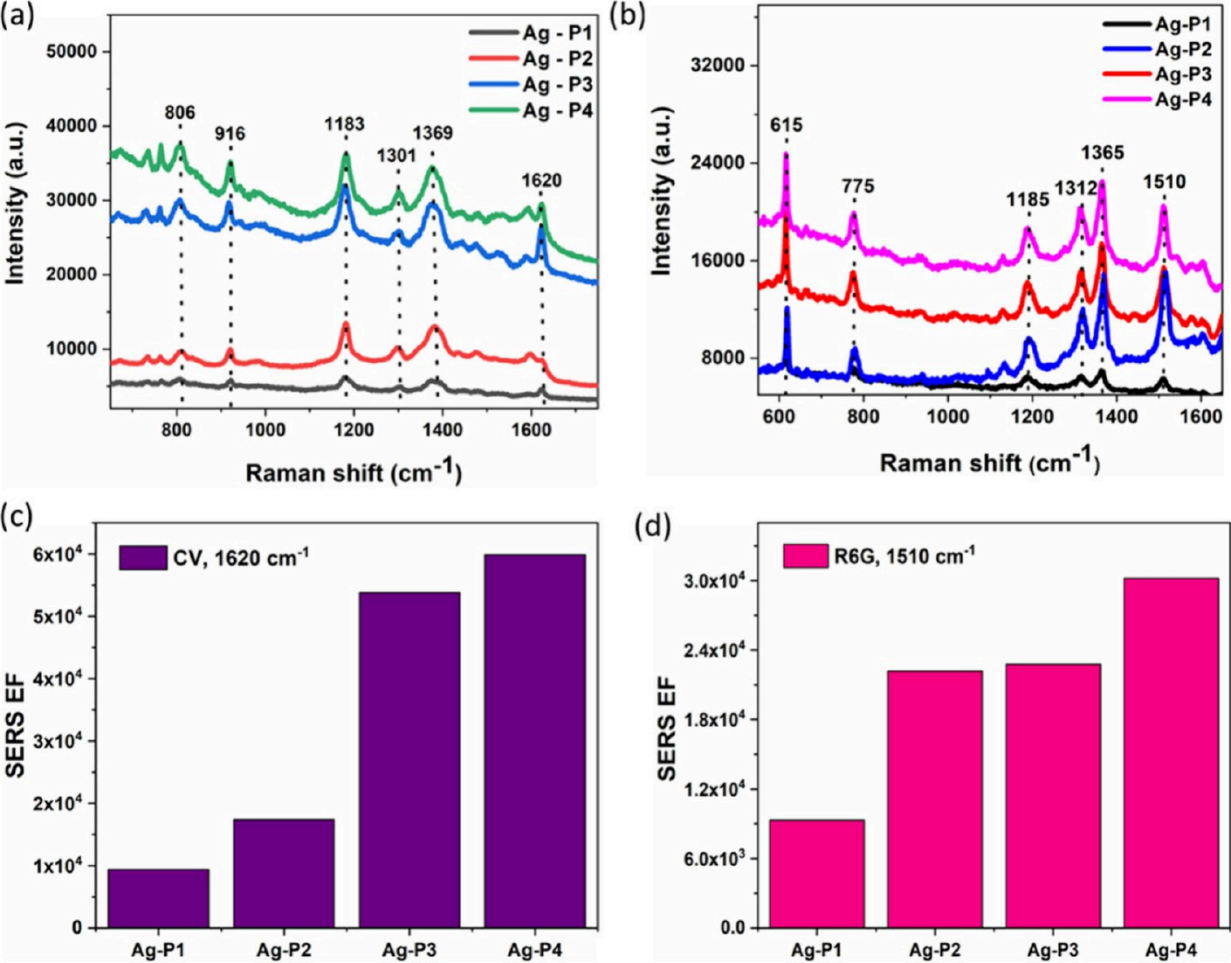
(a, b) Comparison of SERS results of Ag-NP samples prepared under different pressures with 4 A. (c, d) Comparison of enhancement factors for different pressures using 4 A.

The SERS performance of the substrates was first optimized by adjusting the thermal evaporation pressure and the applied current. To test the SERS activity, crystal violet (CV) and rhodamine 6G (R6G) were used as probe analyte molecules by using a 633 nm excitation laser source. The SERS substrates were prepared by depositing the CV (10^–6^ M) or R6G (10^–6^ M) onto each substrate and allowed to naturally dry at room temperature before recording SERS spectra. Figure S7 shows the Raman spectra of CV (10^–6^ M) on the Ag NP substrate obtained by different pressures and applied current during thermal evaporation. As can be seen from Figure S7, all of the spectra of CV on the Ag NP film substrate exhibit the Raman peaks at 806, 916, 1183, 1301, 1369, and 1620 cm^–1^, which are assigned to the characteristics Raman signals of CV molecule.^19^ The higher Raman signal intensity of CV was observed for the substrate obtained at low pressure (2 × 10^–4^ mbar) with the applied current of 4 A (Figure S7). Figure 3a,b shows the SERS spectra of CV and R6G (10^–6^ M) onto the Ag film substrate obtained at different pressures with a constant applied current of 4 A. The highest SERS intensity was observed for the Ag–P4 substrate in the peak of CV at 1620 cm^–1^ and R6G at 1510 cm^–1^, as shown in Figure 3c,d. These results further confirm the significant Raman signal enhancement in the nanoporous structure of Ag NPs (Ag–P4).

To evaluate the SERS performance of the samples, the SERS enhancement factor (SERS-EF) was estimated quantitatively according to the previous work,^19^ by using the following equation:
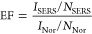
1where *I*_SERS_ and *I*_NOR_ are the intensities of the SERS signals of the test molecule adsorbed on the substrates of Ag NPs and the normal Raman spectrum and *N*_SERS_ and *N*_NOR_ represent the corresponding number of test molecules at the point where the laser is focused. We considered the uniform distribution of molecules on the substrates of Ag NPs. The value of the analyte molecule for the concentrations to obtain the SERS spectrum is 10^–6^M, and the normal Raman spectrum of the test molecules (without substrate) is 10^–3^M. By comparing Raman signal intensities at 1620 cm^–1^ of CV, the SERS EFs were estimated for all of the obtained substrates as shown in Figure 3c. The highest EF value obtained was about 5.4 × 10^4^ for the Ag substrate obtained at low pressure (2 × 10^–4^ mbar) and an applied current of 4 A. To elucidate the influence of nanoporous Ag NP substrates for SERS enhancement, the smooth Ag films were prepared without introducing the DES over a growth substrate and SERS spectra recorded with CV (10^–6^ M) on the substrate (Figure S8). The obtained film does not show any nanoporous structure; however, the films are composed of smaller Ag nanoparticles that are compacted over the entire film. The SERS signal intensity is much lower (∼6-fold lower) than that of the smooth Ag NP film substrate, which confirms the significant role of the nanoporous structure in enhancing the SERS signal intensity. Specifically, the nanoporous 3D structure is capable of enriching the deposited probe molecules within the pores, as well as enhancing the stability and orientation of the adsorbed molecules.^19^

**Figure 4:**
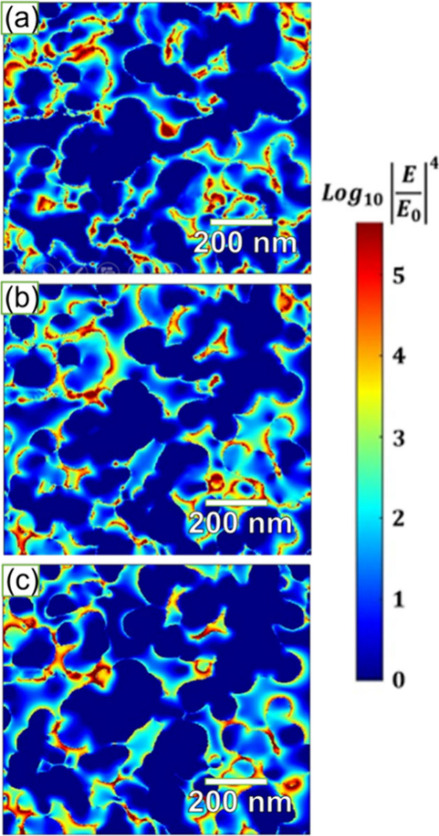
FDTD simulated electromagnetic (EM) field distribution in the nanoporous Ag NPs films (Ag–P4) under laser excitation wavelengths of (a) 532, (b) 633, and (c) 785 nm, respectively.

To confirm the formation of “hotspots”, the electromagnetic (EM) field distribution in the nanoporous Ag NP film was further investigated by finite-difference time-domain (FDTD) simulations. Figure 4a–c show the EM field distribution of nanoporous Ag film with three different irradiation wavelengths such as 532, 633, and 785 nm, respectively. It can be observed that the local electromagnetic (EM) fields are gradually increased with the increasing of irradiation wavelengths, and strong EM fields were observed with irradiation wavelengths of 633 and 785 nm from a laser source (Figure 4b,c). These results revealed that the local EM fields are concentrated between the edges of each nanoporous Ag structure, which serve as “hotspots” of SERS signal enhancement when the analyte molecules are adsorbed in the vicinity of the nanopore sites. Additionally, the irregular 3D nanoporous film features numerous asperities and edges that further enhance EM fields, as previously demonstrated.^4^ Theoretical SERS enhancement factors (SERS-EFs) were estimated to be approximately 2.20 × 10^7^, 2.77 × 10^7^, and 2.46 × 10^7^ for laser irradiation at 532, 633, and 785 nm, respectively. These findings indicate that the maximum EM field distribution was observed with excitation at 633 nm. It is noteworthy that the estimated theoretical EFs are on the order of 10^7^, which is higher than the estimated EFs experimentally (5.4 × 10^4^). This difference could be attributed to variations in the density of CV molecules, changes in pore sizes, and differences in laser incidence sites. Importantly, the enhanced SERS activity and sensitivity of the nanoporous Ag NP films obtained at low pressure correlate directly with higher theoretical SERS EFs, highlighting the critical role of EM field confinement in creating “hotspots” for SERS signal enhancement.

### Signal Sensitivity, Reproducibility, and Stability of a Nanoporous SERS Substrate

**Figure 5:**
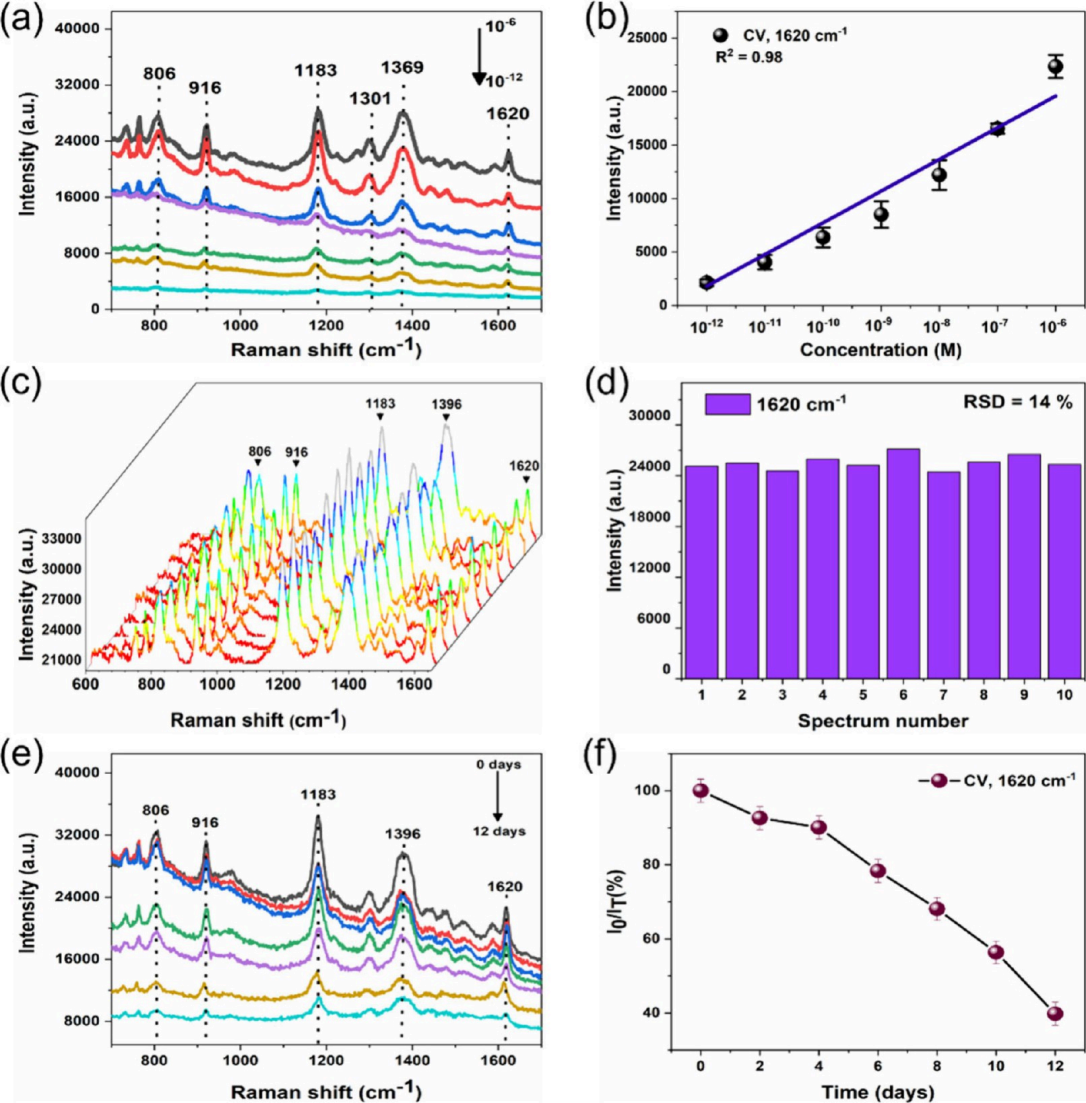
(a) SERS spectrum of the test CV molecules onto a nanoporous Ag NP film substrate with varying CV concentrations between 10^–6^ and 10^–12^ M. (b) Linear calibration corresponding to the Raman peak of 1620 cm^–1^ vs concentrations of CV. (c) SERS spectra of CV (10^–6^ M) recorded from 10 randomly selected spots on the nanoporous Ag NP substrate. (d) Distribution of peak intensity at 1620 cm^–1^ for 10 different spots. (e) Raman spectra recorded for CV (10^–6^ M) for a 12 day period. (f) Corresponding changes of the Raman signal intensity of CV at 1620 cm^–1^.

The porous structural feature of 3D porous Ag NP substrates enables the analyte molecule (CV) to diffuse inside the porous structure, which can significantly enhance the SERS signal intensities. To further verify the sensitivity of a porous Ag NP substrate, the CV concentration was varied in a range of 10^–6^ to 10^–12^ M, and the SERS spectra were collected. As shown in Figure 5a, the SERS spectra of CV with varied concentrations from 10^–6^ to 10^–12^ M have an obvious decrease in their intensities. Specifically, even at a lower CV concentration of 10^–12^ M, characteristic peaks were observed, indicating that the as-fabricated 3D nanoporous Ag NP substrate has a high sensitivity. The limit of detection (LOD) for the models was recalculated according to the following equation:^51^
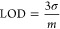
2where σ is the standard deviation of the SERS intensity of the blank, while *m* represents the slope of plotted calibration curves. The determined LOD was about 1.5 × 10^–13^ M for trace detection of CV. Additionally, it can be seen from Figure 5b, that the changes in Raman intensity at the prominent 1620 cm^–1^ peak were plotted as a function of CV concentrations, revealing a linear behavior, which proves that the nanoporous Ag substrate has excellent SERS performance for quantitative SERS detection of analyte molecules with wide concentration ranges.

The substrate reproducibility and storage stability are considered to be important parameters of SERS-active substrates for their applications on various platforms. Therefore, the SERS reproducibility was examined by point-to-point Raman scanning. Figure 5c shows the Raman spectrum of 10 random points on the same substrate. The SERS intensities for the 10 spectra obtained are highly uniform, indicating that the SERS substrate is highly reproducible and stable. To quantitatively assess SERS reproducibility, we estimated the relative standard deviation (RSD) of a prominent peak at 1620 cm^–1^ from 10 SERS spectra (Figure 5d). The estimated RSD value is about 14%, which suggests good SERS reproducibility. The performance of the SERS substrate can also be determined by its stability for a long period of time without losing its functionality. To further examine the stability of the nanoporous Ag NP substrate, the SERS spectra of CV molecules (10^–6^ M) over the substrate and the SERS spectra every 2 days for a 2 day period under ambient environment storage conditions were acquired (Figure 5e). As seen in Figure 5f, after being stored for 12 days in an ambient environment, the Raman intensity of the peak at 1620 cm^–1^ is reduced by about 60% from the initial intensity value of the freshly prepared SERS substrate. Such a reduction of stability could be due to the formation of a Ag oxide layer over the nanoporous Ag NP substrate during environmental exposure.

### SERS Detection of PET Nanoplastics Using Nanoporous Ag Substrate

**Figure 6:**
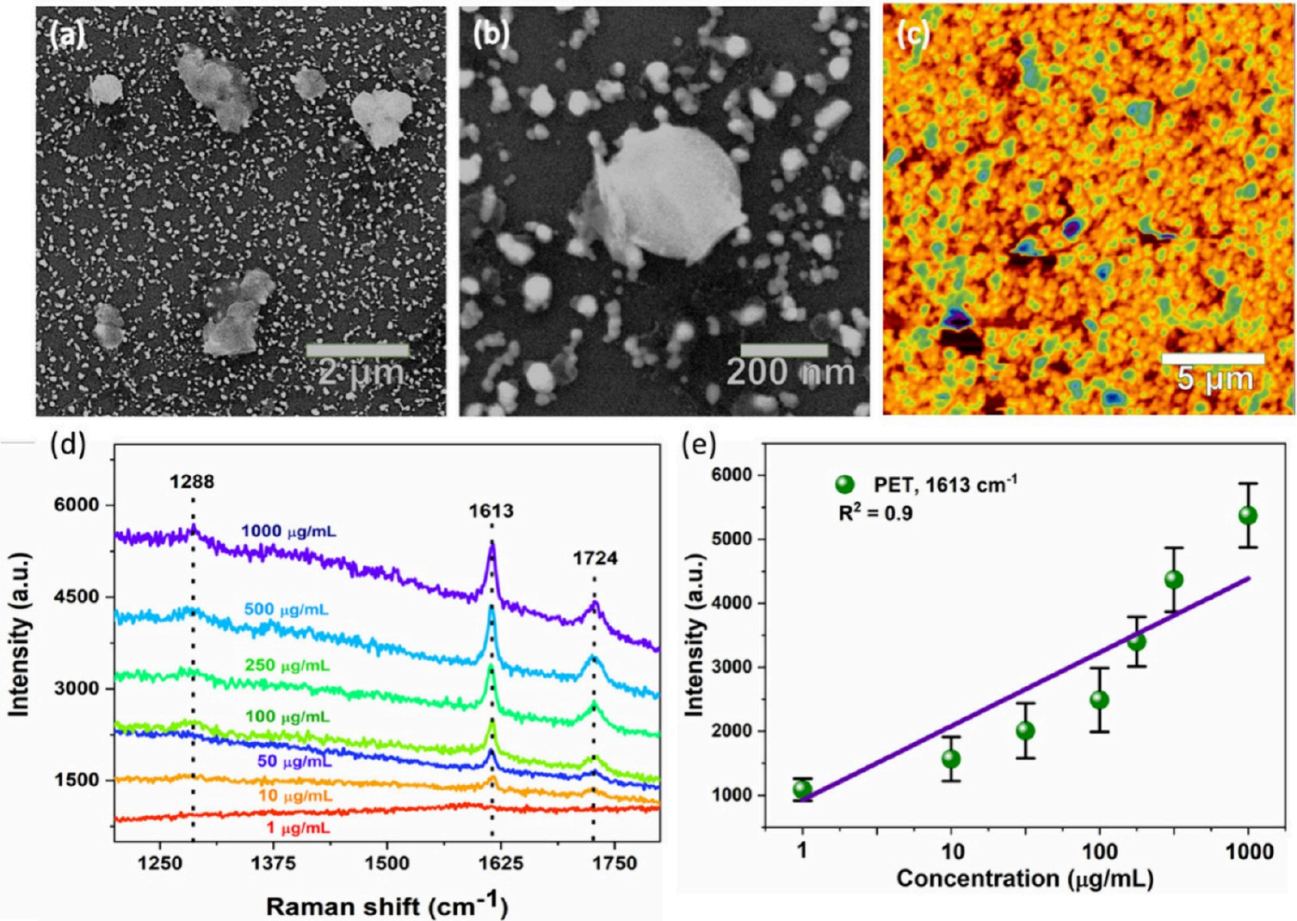
Morphological characterization of PET nanoplastic particles over the nanoporous Ag-SERS substrate. (a) Low and (b) high magnification FE-SEM. (c) AFM-topographic images of the PET nanoplastics over the SERS substrate. (d) SERS spectra of PET nanoplastic particles with varying concentrations. (e) Relationship between PET concentration vs SERS peak intensity was 1613 cm^–1^.

To further demonstrate the practical application of the SERS substrate, we investigated the SERS detection of PET nanoplastics. The PET nanoplastics with a size of 200 nm (Figure S9) were synthesized by following our earlier report.^45^ The PET nanoplastics with varied concentrations were dispersed in DI water and drop-cast over the nanoporous Ag substrate and dried in a natural environment for SERS measurement. As shown in Figure 6a–c, the SEM and AFM images of the PET nanoplastics over a SERS substrate confirmed that the PET nanospheres were trapped inside the nanoporous structure of Ag. The average particle size of the PET nanospheres was estimated to be about ∼200 nm. Figure 6d,e show the SERS spectra of the PET nanospheres with varying concentrations deposited onto the nanoporous Ag NP substrate and the corresponding linear calibration plot showing the Raman intensity as a function of concentration. The SERS spectra exhibit characteristic peaks at 1288, 1613, and 1724 cm^–1^, corresponding to the PET particles.^52^ Specifically, the peak at 1288 cm^–1^ is assigned to the CH_2_ twisting vibrations of aromatic in-plane CH deformation.^53^ The characteristic peaks at 1613 and 1724 cm^–1^ are attributed to the C–O and C=O stretching vibrations, respectively.^54^ The SERS signal was detected even at a concentration of 1 μg/mL, suggesting that the SERS substrate exhibits excellent sensitivity toward nanoplastics. The LOD was calculated to be about 0.38 μg/mL. The calibration plot (Figure 6e) indicates that there is a good linear relationship between the SERS intensity at 1724 cm^–1^ and the PET concentrations with *R*^2^ = 0.92, suggesting the substrate is capable of quantitative detection of PET nanoplastic particles. The high sensitivity of the Ag–P4 substrate could be attributed to the PET nanoplastic particles with a size of 200 nm being able to effectively diffuse within the nanopore (size 302.1 ± 8.8 nm) as well as strong EM field distribution when compared with the other substrates (Figures S5 and S6), resulting in high sensitivity even at low concentrations of PET nanoplastic particles.

**Figure 7:**
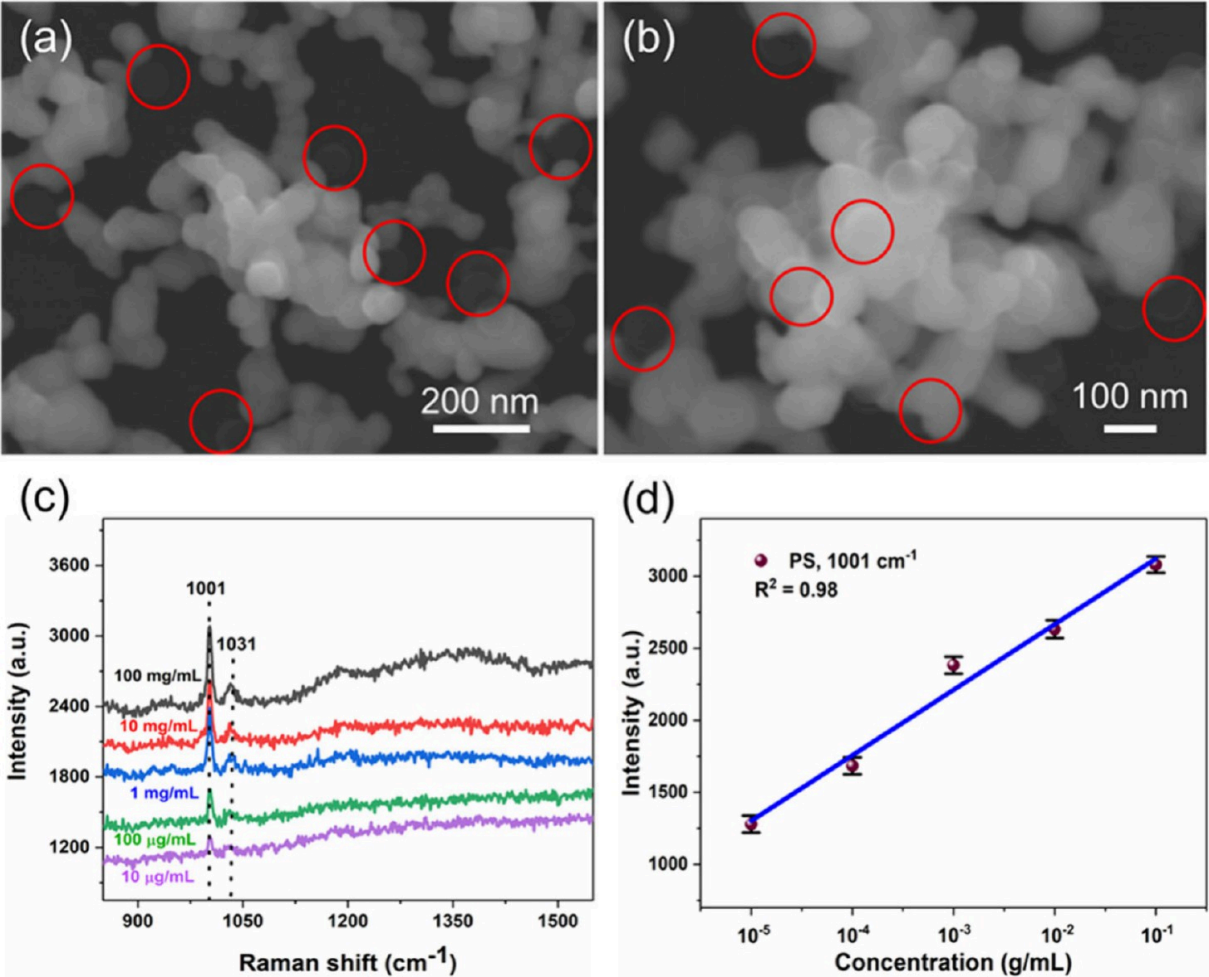
(a, b) FE-SEM images of PS nanospheres (size of 100 nm) over the nanoporous Ag NP film substrate. (c) SERS spectra of PS with different concentrations and (d) relationship of PS concentration as a function of SERS peak intensity at 1001 cm^–1^.

To evaluate the SERS detection of another type of nanoplastics, we have also investigated the detection of commercial polystyrene nanospheres (PS, size ∼100 nm) using a nanoporous Ag substrate (Figure 7). As shown in the SEM images (Figure 7a,b), the PS nanospheres with a size of about 100 nm are trapped inside the nanoporous Ag NP structures as marked in the red circles. Figure 7c shows the Raman spectra of PS particles showing the distinguished peaks at 1003 and 1031 cm^–1^, which are associated with the ring mode vibrations of monosubstituted aromatic compounds or C–C ring breathing and C–H vibration in PS nanospheres.^55^ These results demonstrated that the Raman signal intensity is found to decrease by decreasing the PS nanospheres concentration, and well-distinguishable peaks were observed even at 10 μg/mL, suggesting the appreciable sensitivity in detection of PS nanospheres. The LOD is estimated to be about 9.8 × 10^–7^ g/mL. The linear trend was obtained for the concentration of PS as a function of the signal intensity of PS at 1001 cm^–1^, indicating the capability of detection over a wide concentration range. It should be mentioned that the signal intensity of PS nanoplastics is weaker compared with the PET nanoplastics, which could be because of the lower Raman scattering cross-section of PS nanoplastics as well as smaller particle size.^55^ These results indicate that the proposed method enables monitoring of another type of nanoplastic with excellent sensitivity for trace analysis of nanoplastics in water.

### Analysis of Nanoplastics in Environmental Water and Diluted Milk Samples

**Figure 8:**
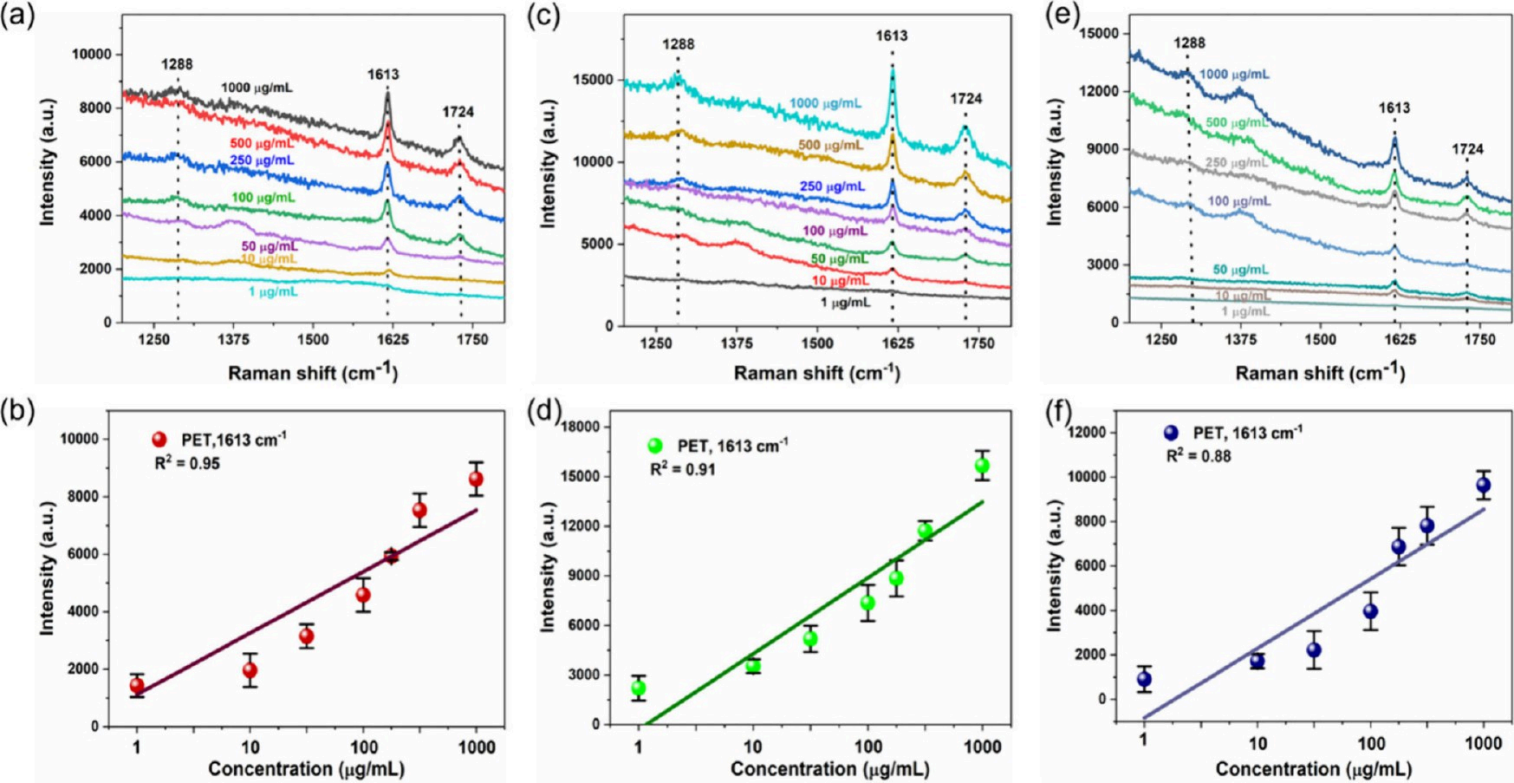
SERS spectra of PET nanoplastics in real environmental water and milk samples and the corresponding linear calibration plot between Raman intensity vs concentration. (a,b) Tap water, (c,d) lake water, and (e,f) diluted milk samples, respectively.

Previous reports of nanoplastics evidence in real samples such as human blood, human lungs, beverages, drinking water, milk, and food packages were observed.^58−61^ Most of the previous studies have found that majority of the food samples contain micro/nanoplastics with a size range from 5 mm to 700 nm, where PET and PS are the most abundant.^59^ Thus, to test the feasibility of the matrix detection of PET nanoplastics in actual environmental water samples, we evaluated the SERS detection of PET nanospheres in tap water and lake water samples. Moreover, to further validate the analytical ability in the detection of PET nanospheres, we have tested the detection in milk samples by diluting about 10 times using DI water. For SERS detection, the PET nanospheres of different concentrations were spiked in tap water, lake water, and diluted milk and then drop-casted on the surface of a nanoporous Ag NP substrate to be allowed to dry overnight naturally. Figure 8a–f displays the SERS spectra of PET nanospheres of different concentrations in tap water, lake water, and diluted milk and the corresponding calibration plot between Raman intensity at 1724 cm^–1^ vs concentrations. The results confirmed that the Raman peaks of PET nanospheres were detected in all three real samples such as tap water, lake water, and diluted milk; the SERS intensity of the peaks was decreased as a function of lowering the PET concentrations. The LOD of PET nanoplastic particles was determined to be about 1.9, 2.7, and 8.4 μg/mL, respectively, in the tested real samples. In addition, the calibration plot of the SERS intensity of PET at 1724 cm^–1^ as a function of concentrations was found to be quite linear in all three real samples, suggesting the excellent detection capability of the SERS substrate in a complex environmental sample and indicating the capability of quantitative SERS detection of PET nanoplastics in environmental samples.

**Figure 9:**
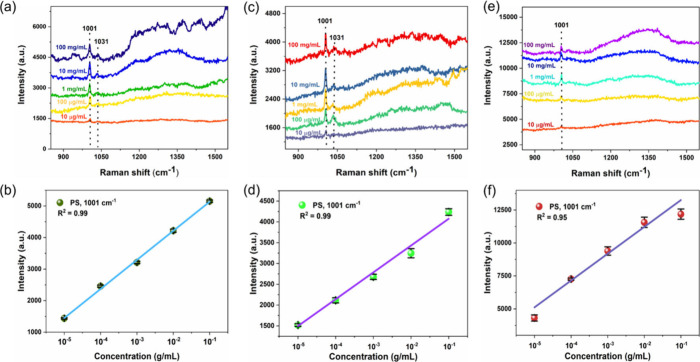
SERS spectra of PS nanoplastics in real environmental water and diluted milk samples and the corresponding linear calibration plot between Raman intensity vs PS concentration. (a, b) Tap water, (c, d) lake water, and (e, f) diluted milk samples.

Figure 9 shows the SERS spectra of PS nanospheres with varied concentrations in three different real samples such as tap water, lake water, and diluted milk, respectively. It is clear from Figure 9a, c, and e that the SERS spectra showed two distinguishable peaks of PS nanospheres at 1001 and 1031 cm^–1^, and the signal intensity decreases when lowering the concentration. The linear response was observed in the PS concentration as a function of Raman signal intensity at 1001 cm^–1^ for all three measured real samples, indicating excellent sensitivity (Figure 9b,d,f). Importantly, even at low concentrations (10 μg/mL), the Raman signals were detected. The LOD was estimated to be about 4.9, 6.3, and 7.5 μg/mL for tap water, lake water, and diluted milk, respectively. This result indicates that the SERS substrate is highly sensitive to the detection of PET and PS nanoplastics in complex environmental samples. As shown in Table 1, the performance of the proposed SERS-substrate for nanoplastics detection is comparable or greater compared with the several recently reported SERS substrates in terms of limit of detection. Such an excellent analytical detection capability of our SERS substrate can be directly correlated to the detection of nano- and microplastics in real-time analysis in a variety of actual environmental water and food samples.

**Table 1:** Comparison of the SERS Detection of Nanoplastics Using Nanoporous Ag NP Film with Previous Literature

SERS substrate	type of nanoplastics	nanoplastics size (nm)	LOD (μg/mL)	ref
Ag/ZnO@ PDMS	polystyrene	800	25	Zhu et al.^54^
bifunctional Ag nanowire membrane	polystyrene	50	1000	Yang et al.^55^
3D-AAO/MoS_2_/Ag	polystyrene	100		Li et al.^18^
AuNSs@ Ag@ AAO	polystyrene	400	50	Le et al.^56^
Ag–Au NPs film	PET	200	10	Carreón et al.^44^
Ag NP@MgSO_4_	polystyrene	50	100	Zhou et al.^57^
nanoporous Ag NPs film	PET, PS	200 nm	0.38	this work
		100 nm	0.98	

## Conclusion

In conclusion, we have fabricated the nanoporous Ag NP-film-based SERS substrate using a green and low-cost synthetic approach by successfully integrating the DESs into the vacuum thermal evaporation process. The results revealed that the controlled self-assembly of aggregated Ag NPs and porosity were grown on the surface of DES-coated substrates, which enabled the formation of a maximum number of “hotspots” for SERS detection. Owing to the nanoporous structure and high density of the “hotspots”, the analyte molecules can diffuse inside the pores, which resulted in significant enhancement of SERS activity for the detection of probe molecules (CV) with an LOD able to reach up to 1.5 × 10^–13^ M, excellent reproducibility, and stability of the substrate. Most importantly, the fabricated substrate has been applied for SERS detection of nanoplastic particles such as PET and PS nanospheres with excellent sensitivity with a LOD as low as 0.38 and 0.98 μg/mL, respectively. Furthermore, the obtained SERS substrate can detect PET and PS nanoplastics in tap water, lake water, and diluted milk samples with excellent sensitivity. Given the exceptional SERS performance of the nanoporous Ag NPs film substrate obtained using the green chemistry approach, it will pave the way for the development of effective SERS sensors with 3D “hotspots” for quantitative detection of a variety of toxic contaminants in the environmental samples.

## Supporting Information Available

SEM Ag NP films obtained at different pressures and applied current, SEM and AFM images of Ag film, SERS spectra of varied Ag substrates immobilized with R6G, and comparison of SERS spectra of different substrates (PDF)
