# Supplementary material for: A Scalable Synthesis of Ag Nanoporous Film As an Efficient SERS-Substrates for Sensitive Detection of Nanoplastics
Source: Langmuir. 2024 Aug 5;40(33):17476–88. doi: 10.1021/acs.langmuir.4c01671 (PMC11340027; doi:10.1021/acs.langmuir.4c01671)
Supplement: Supplementary file 1 — la4c01671_si_001.pdf [file la4c01671_si_001.pdf]

Supporting information for:

A scalable Synthesis of Ag Nanoporous film as an efficient SERS-Substrates for Sensitive Detection of Nanoplastics

Rafael Villamil Carreón, [†] Ana G. Rodríguez-Hernández, [‡] Laura E. Serrano de la Rosa, [Δ] Ma. Estela Calixto, [Δ] J.J. Gervacio-Arciniega, [*#] Siva Kumar Krishnan.[§*]

[†]Facultad de Ciencias Físico Matemáticas, Benemérita Universidad Autónoma de Puebla, Av. San Claudio y Av. 18 sur., Puebla, Pue., C. P. 72570, México.

[‡]CONAHCyT-Centro de Nanociencias and Nanotecnología, Universidad Nacional Autónoma de México, Km 107 Carretera Tijuana-Ensenada Apdo Postal 14, CP. 22800 Ensenada, B.C. México.

[Δ]Instituto de Física, Benemérita Universidad Autónoma de Puebla, Av. San Claudio y Blvd. 18 Sur, Col. San Manuel, Ciudad Universitaria, Puebla, Pue 72570, México

[#]CONAHCyT- Facultad de Ciencias Físico Matemáticas, Benemérita Universidad Autónoma de Puebla, Apdo. Postal J-48, Puebla 72570, México.

[§]CONAHCyT- Instituto de Física, Benemérita Universidad Autónoma de Puebla, Av. San Claudio y Blvd. 18 Sur, Col. San Manuel, Ciudad Universitaria, Puebla, Pue 72570, México.

Corresponding authors:

JJG: jjgervacio@hotmail.com

SKK: sivakumar@ifuap.buap.mx

Table of content

Figure S1. SEM images of Ag NPs films obtained by thermal evaporation of Ag onto DES-coated substrates with different pressure, and applied current……………………………………………………………………………………S3.

Figure S2. The size distribution histogram of Ag NPs obtained at different deposition pressures…………………………………………………………………………...S4.

Figure S3. SEM and AFM topographical image of Ag film deposited over glass substrate without coating DES on it………………………………………………..S5.

Figure S4. SEM images of Ag NPs films obtained using different types of DES components…………………………………………………………………………...S5.

Figure S5: AFM topographic, line profile and corresponding pore size distribution histogram of the nanoporous Ag NP film obtained at low pressure……………….S6.

Figure S6. AFM topographic images and corresponding line-profile and pore size distribution histogram of the Ag NPs films obtained at different pressures……….S6.

Figure S7. Comparison of SERS Spectra of Ag-NPs film prepared under different pressures and applied currents and corresponding SERS EFs values..................S7.

Figure S8. Comparison of the SERS activity of CV (10^{-6} M) on nanoporous Ag NP substrate with Ag NP film and bare CV molecules on glass substrate……………S8.

Figure S9. SEM images and Dynamic light scattering (DLS) spectrum of PET nanoplastics particles dispersed in DI water.,……………………………………...S9.

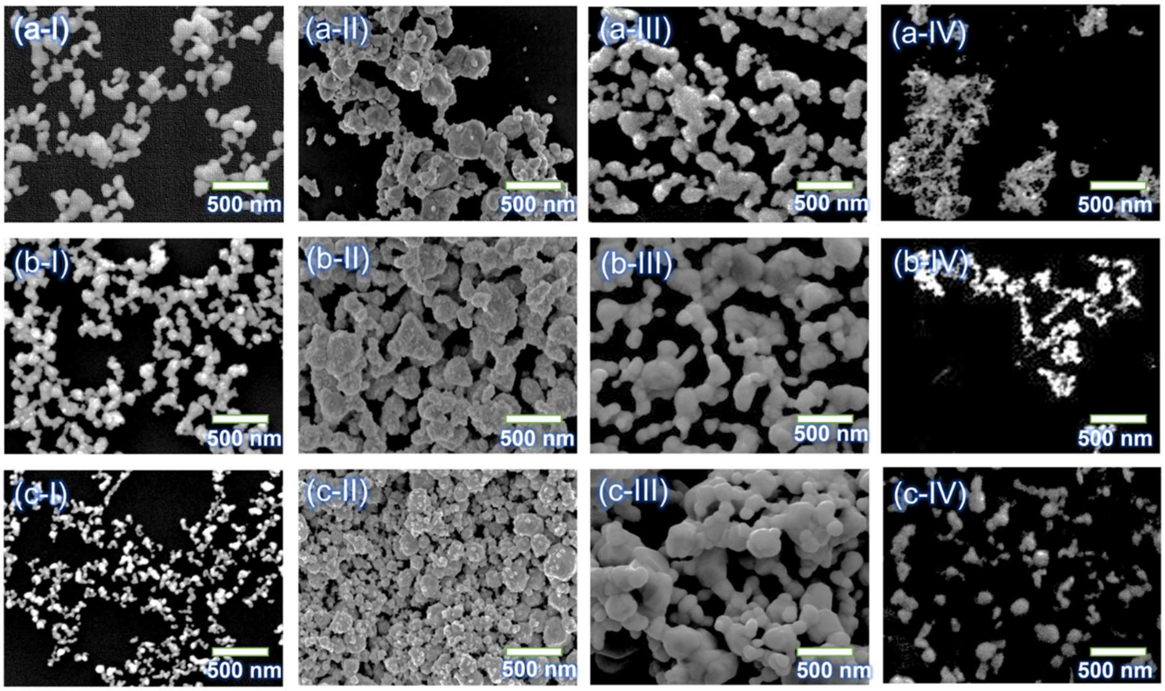

Figure S1. SEM images of Ag nanoparticles films obtained by thermal evaporation over DES (ChCl: U, ratio molar 1;2) with different pressures with P1 ((a-I) 2amps, (a-II) 4 amps, (a-III) 8 amps, (a-IV) 12 amps), P2 ((b-I) 2 amps. (b-II) 4 amps, (b-III) 8 amps, (b-IV) 12 amps), P3 ((c-I) 2 amps, (c-II) 4 amps, (c-III) 8 amps, (c-IV) 12 amps).

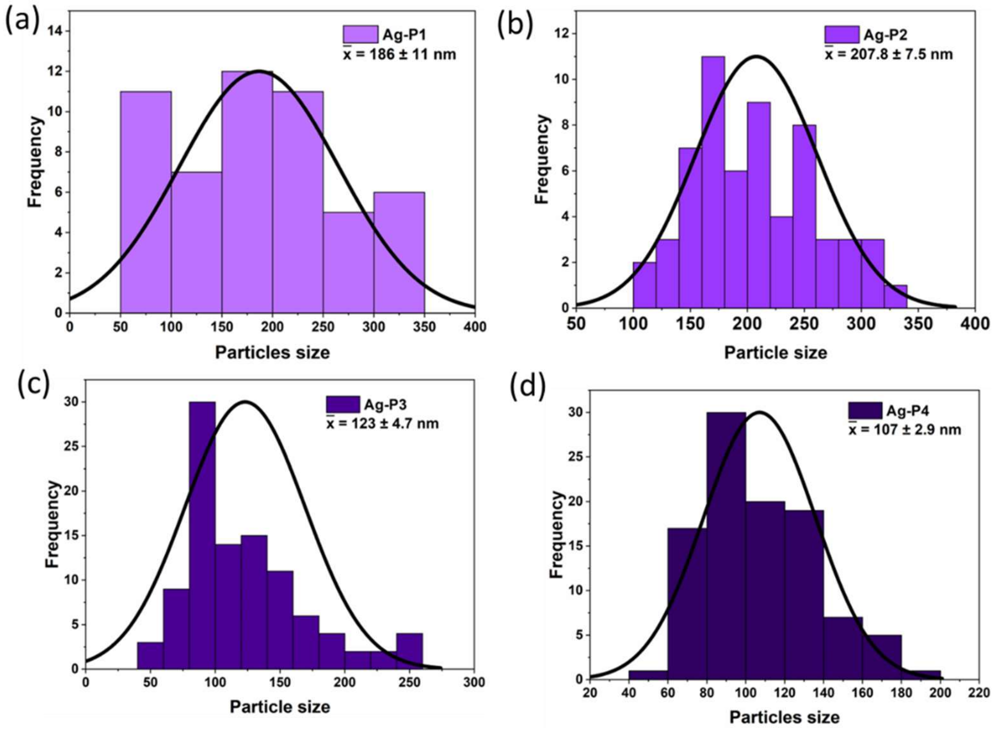

Figure S2. (a-d) Size distribution histogram of Ag NPs films obtained at different deposition pressures. To obtain the average particle size approximately 80-100 particles were counted.

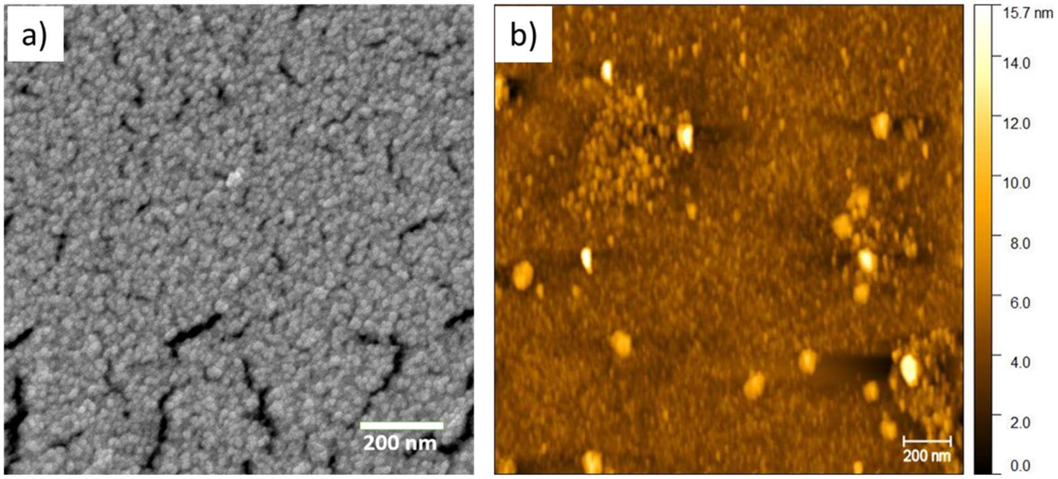

Figure S3. Morphological characterization of Ag films deposited over glass substrate without coating DES on it. a) SEM and b) AFM topographical image of Ag film.

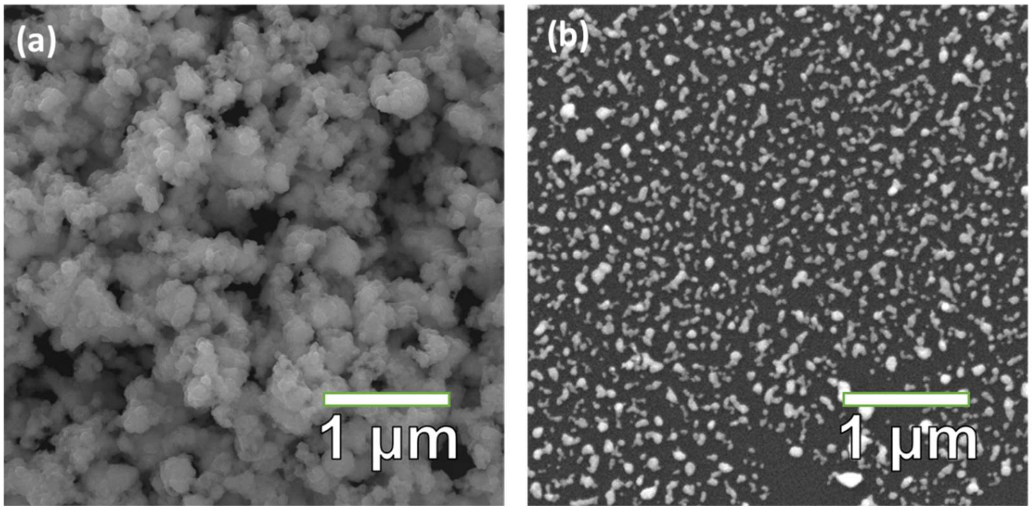

Figure S4. Typical SEM images of Ag NPs films obtained using (a) ChCl: malonic acid (molar ratio 1:1), (b) ChCl: Ethylene glycol (molar ratio of 1:4), coated substrates.

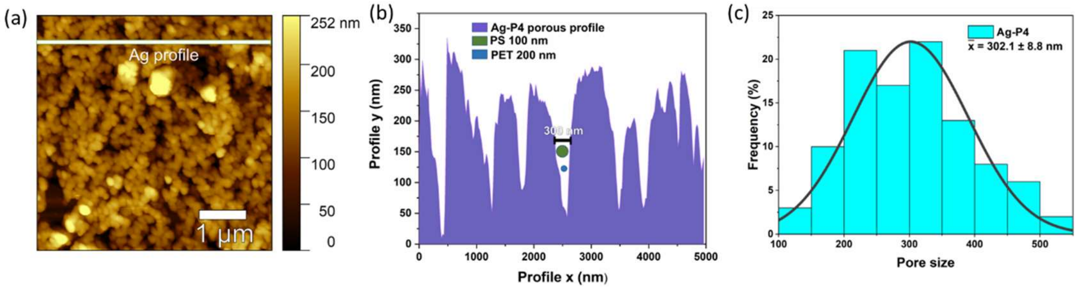

Figure S5: a) AFM topographic image of nanoporous Ag NPs film, b, c) Line profile and corresponding pore size distribution histogram of the nanoporous Ag NP film.

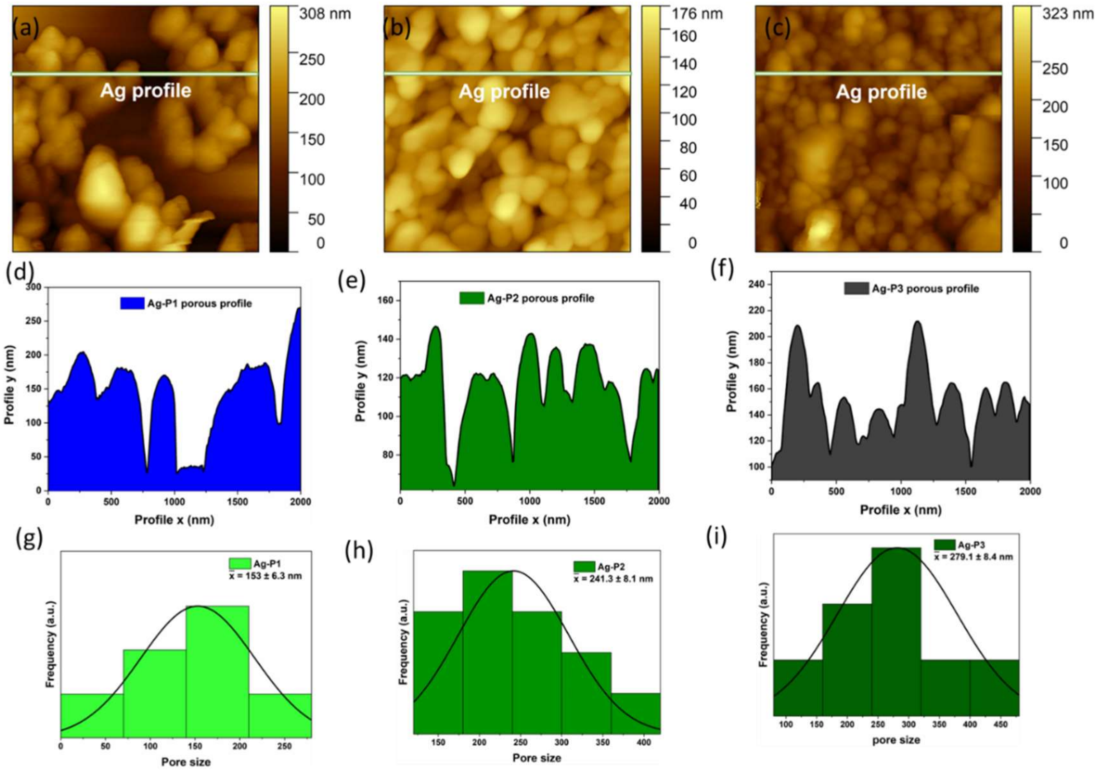

Figure S6. (a-c) AFM topographic images, (d-f) corresponding line profile and (g-i) pore size distribution histogram of the Ag NPs films obtained at different pressures (Ag-P1, Ag-P2, and Ag-P3 samples).

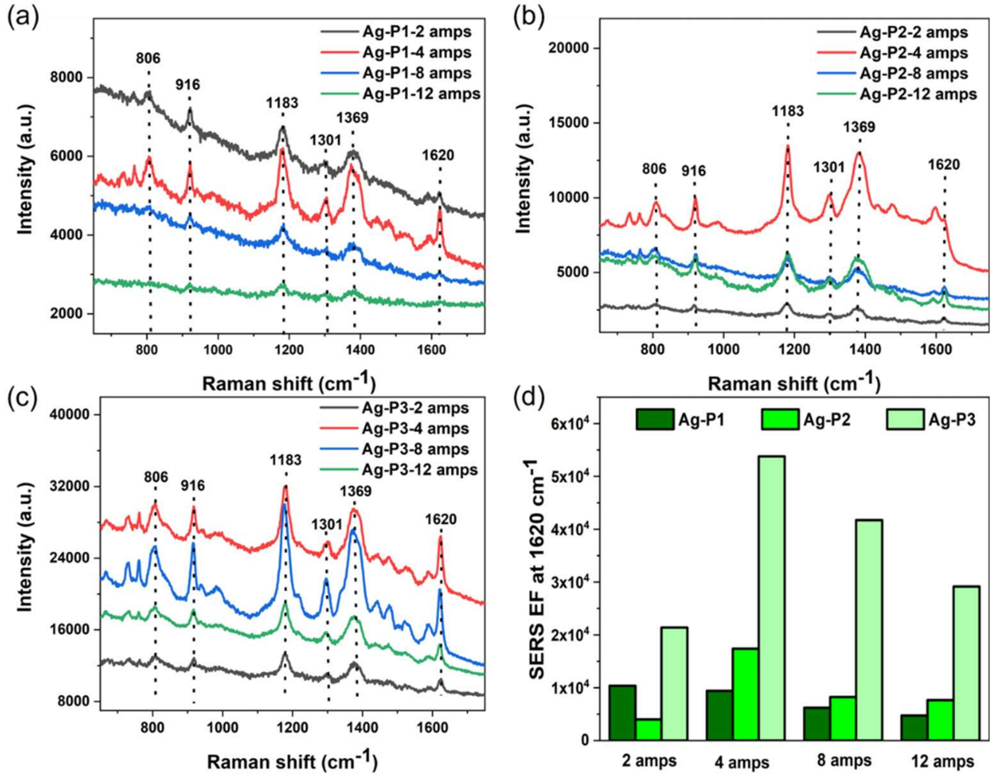

Figure S7. Comparison of SERS Spectra of Ag-NPs film prepared under different pressures and different currents. (a) Ag-P1, (b) Ag-P2, (c) Ag-P3, (d) Comparison of enhancement factors (EFs) of the SERS substrates obtained under different pressures using different deposition currents.

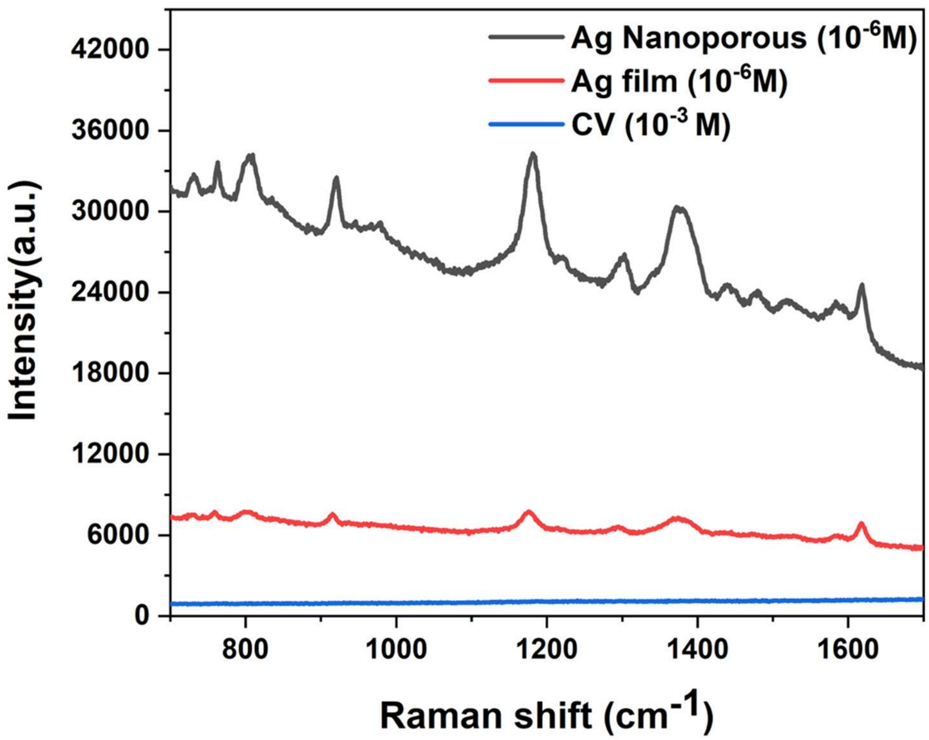

Figure S8. Comparison of the SERS spectra of CV (10^{-6} M) on nanoporous Ag NP substrate with bare Ag NP film and bare CV molecules on glass substrate, respectively.

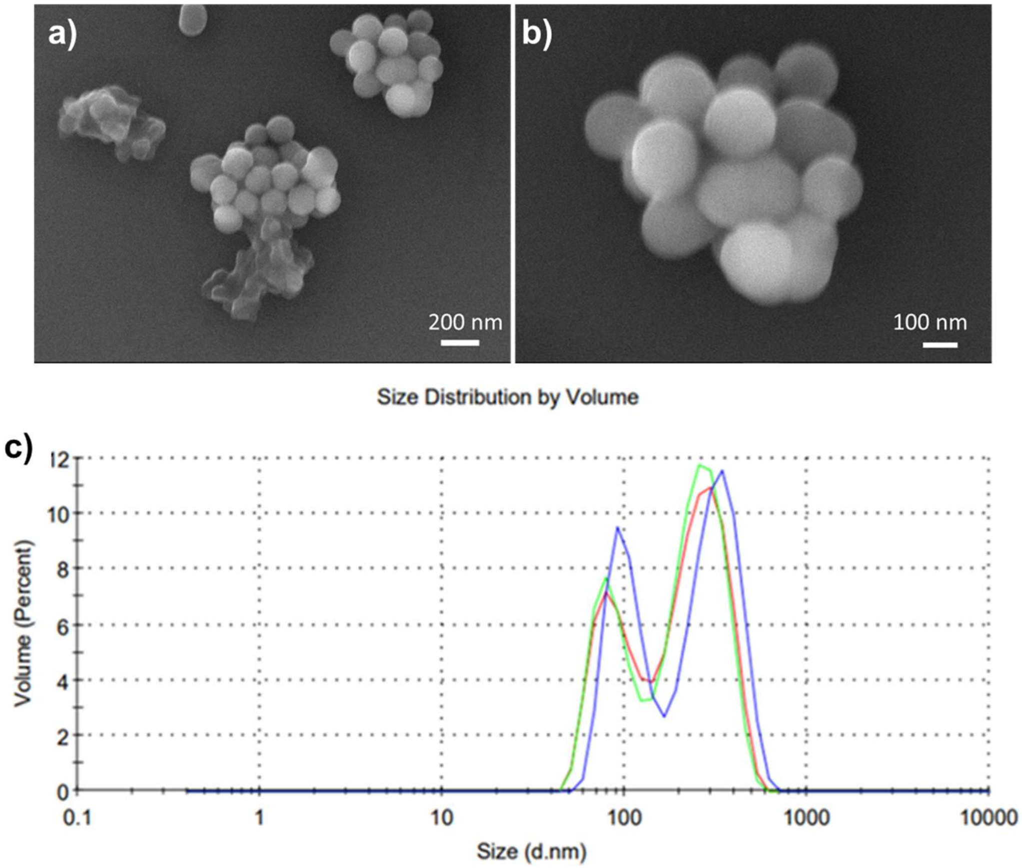

Figure S9. Characterization of PET nanoplastics particles. (a, b) SEM images of PET nanoplastics particles, (c) Dynamic Light scattering (DLS) spectrum of PET Nanoplastics particles dispersed in DI water.
